# Exploring the Influence of Chalcogens on Metalloporphyrins: A DFT Study

**DOI:** 10.3390/molecules30112254

**Published:** 2025-05-22

**Authors:** Beenish Bashir, Andre Z. Clayborne

**Affiliations:** 1 Department of Chemistry and Biochemistry, George Mason University, Fairfax, VA 22030, USA or bbashir2@gmu.edu; 2 Quantum Science and Engineering Center, George Mason University, Fairfax, VA 22030, USA

**Keywords:** porphyrins, spin, quantum, density functional theory

## Abstract

Metalloporphyrins and porphyrins (MPs) have garnered increasing attention as potential candidates for molecular-based electronic devices and single-atom catalysis. Recent studies have found that electronic structure calculations are important factors in controlling the performance of MPs as building blocks for single-molecule devices. Our study investigates metalloporphyrins with central 3d-metals from Sc to Cu and chalcogen containing anchoring groups such as -SH, -SeH, and -TeH substituted at the meso-position of the porphyrin rings. We carried out Density Function Theory (DFT)-based calculations to determine the ground state geometry, spin multiplicity, spatial distribution of the molecular orbitals, and electronic structure descriptors to gain insights into the reactivity trends and possible impact on factors influencing electron transport properties. The results suggest that the central metal shapes the spin multiplicity, while variations between sulfur, selenium, and tellurium play a role in charge distribution. This study provides insights into how the selection of the central metal and control of spin channels influence the electronic structure and reactivity of metalloporphyrin molecules. The knowledge provided here can play a role in the design of porphyrin-based molecular materials for diverse applications in molecular junctions, catalysis, photovoltaics, and sensing.

## 1. Introduction

The porphyrins and their derivatives have long intrigued researchers due to their dual significance in biological systems and various technological applications [1,2,3]. These molecules serve as essential components in enzymes, playing vital roles in biological processes. Additionally, porphyrins exhibit diverse technological applications across fields such as catalysis, photovoltaics, and sensing, making them subjects of extensive research and interest [4,5]. It has been reported that metalloporphyrin-based molecular junctions can exhibit half-metallic spin-polarized transport properties suitable for spintronic applications [6].

While the majority of studies have focused predominantly on metal porphyrins, with iron taking center stage, the exploration of transition metal-doped complexes has been somewhat limited [7,8]. This research gap is particularly noticeable in our understanding of the electronic structures of metalloporphyrins, especially those involving first-row transition metals beyond Fe/Ti/Ni [9,10,11,12].

The central metal governs the intricate chemistry of metalloporphyrins at the core and the carefully chosen peripherally and axially fixed substituents [13,14,15]. These substituents significantly influence the electronic density distribution within the macrocycle, consequently dictating the reactivity and stability of the compound. Despite the extensive study of porphyrins and their derivatives, there remains a notable gap in understanding the transition metal-doped complexes, presenting a compelling avenue for investigation.

It has been reported that -SH forms strong covalent bonds with metal surfaces (e.g, gold), leading to better electronic coupling between the iron porphyrin molecule and the electrodes. This strong coupling facilitates efficient charge transport through the molecule, resulting in higher conductance [16]. The spin multiplicity and the distribution of spin-up and spin-down electrons in 3d-transition metalloporphyrins are critical factors in determining the electron transport properties and rectification behavior of molecular junctions. Understanding and controlling these spin-related properties enable the design of molecular devices with tailored functionalities, including enhanced rectification, spintronic applications, and magnetoresistance effects. That is the challenging part that needs to be addressed in molecular-based metalloporphyrin devices, such as molecular rectifiers, molecular junctions, transistors, switches, or wires.

In our previous study, we delved into the role of central transition metals and anchoring groups in customizing electron transport using single molecules, focusing on Ni- and Zn-based metalloporphyrins (NiP and ZnP) with a range of anchoring groups (X = -SH, -SeH, -TeH, -F, -Cl, -Br, -CN, -NH_2_, -PH_2_) [17]. The present work undertakes a comprehensive Density Functional Theory (DFT) study of the electronic structures, bonding in a series of 3d-transition metal porphyrins, and spin multiplicity channels. Our focus narrows to meso-substituted anchoring groups -SH, -SeH, and -TeH in 3d-transition metals (Sc--Cu) diphenyl metalloporphyrins, encompassing the first-row transition metals. Despite extensive research, the intricate interplay between the anchoring groups, particularly -SH, -SeH, and -TeH surrounding the porphyrin macrocycle and the nature of the first-row transition metal center with different spin channels, remains a complex puzzle. This study aims to unravel this complexity and provide insights into the delicate interplay between these factors. This research offers crucial insights into the designing of novel porphyrin-based materials, providing a foundation for tailoring their electronic properties for applications in molecular junctions, catalysis, photovoltaics, sensing, and beyond.

## 2. Results

### 2.1. Metalloporphyrins Composition

**Figure 1:**
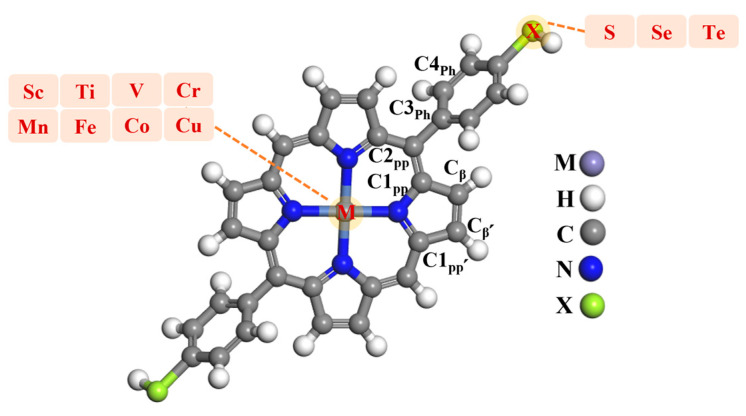
Metal diphenyl porphyrin, where M represents the central metals (M = Sc--Cu), while X represents anchoring groups (-SH, SeH, and TeH).

Figure 1 provides the example composition of the metalloporphyrins (MPs) in this study. The phenyl groups were placed at the meso-positions of the porphyrin, with a transition metal atom at the center. Different functional groups (e.g, -SH, -SeH, and -TeH) were incorporated at the end of the phenyl group, these are usually referred to as the anchoring group, which are labeled in Figure 1. All metal diphenyl porphyrin derivatives have a special, entirely symmetrical structure, with the main body structure being porphyrin.

### 2.2. Structural Analysis of Metalloporphyrins with Thiol Termination

The incorporation of transition metals with varying occupancies of *d*-orbitals between 3d^1^ and 3d^10^ enables the investigation of MDPPSH with different spin states, ranging from singlet to quintet as shown in Table S1 in Supporting Information. Spin multiplicity and the number of unpaired electrons in a molecule significantly impact the transport properties of MPPs [18]. They influence electron transfer rates, redox potential matching, and even Förster Resonance Energy Transfer (FRET) efficiency in some cases [19,20]. Understanding this influence allows us to design and engineer metalloporphyrins for enhanced performance in molecular transport applications. Our investigation into the ground states of 3d-MPP revealed the presence of both low-spin and high-spin configurations. Notably, for elements like V, Cr, and Mn, the calculations in Table S2 of Supporting Information indicate that high-spin states possess the lowest energy, making them the ground state for these specific metalloporphyrins.

**Figure 2:**
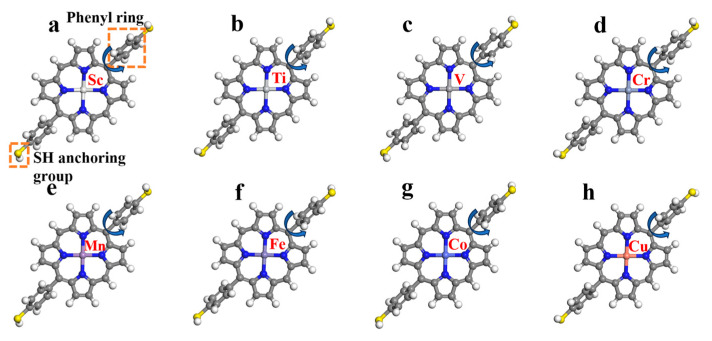
Optimized structures of metals-based diphenyl porphyrin with sulfur (MDPPSH) (ScDPPSeH (**a**), TiDPPSH (**b**), VDPPSH (**c**), CrDPPSH (**d**), MnDPPSH (**e**), FeDPPSH (**f**), CoDPPSH (**g**), CuDPPSH (**h**) systems, respectively). The arrows around the phenyl rings indicate tilting of phenyl ring with respect to PP core.

Figure 2 shows the optimized structures of MDPPSH with various central metals (Sc--Cu). The MDPPSH molecules have a nearly planar macrocycle (porphyrin core). Within the macrocycle, the bond lengths vary slightly (Table 1) depending on the central metal. This suggests that the electronic distribution of the central metal affects the geometry of the molecule, which can be attributed to interactions between the metal d-orbitals and the porphyrin core. With increasing metal size, trends observed in bond lengths indicate stronger binding between the metal and the porphyrin when moving from Sc to Cu. The C-C bond lengths within the porphyrin ring fall between single and double bonds due to the extended π-electron conjugation. The good agreement between the M-N bond lengths obtained in our calculations (Table S3 of Supporting Information) and those reported in the literature validates our computational methodology.

Despite the planar macrocycle, steric constraints twist the phenyl rings out of the plane. The extent of this twisting (quantified by dihedral angles) varies depending on the central metal. Among the studied MDPPSH with different central metals, the CoDPPSH molecule exhibits the most significant tilting of the phenyl rings, while the TiDPPSH molecule has the least tilted phenyl rings (Table 1). This suggests a crucial role of central metal in dictating the orientation of the phenyl groups. The specific electronic configuration of Co in CoDPPSH likely leads to stronger interactions with the phenyl rings, causing them to twist out of plane to a greater extent compared to Ti in TiDPPSH (Table 1). This variation in phenyl ring orientation could potentially be exploited for designing MDPPSH-based materials with tailored properties for various applications, spanning from electronics to biomedicine and energy storage [21,22].

### 2.3. Electronic Structure Properties

**Figure 3:**
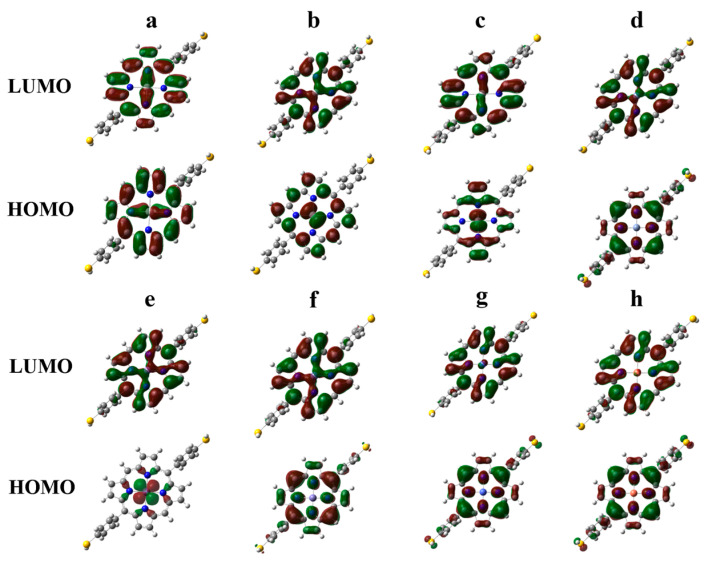
Spatial distributions of HOMO and LUMO calculated for ScDPPSH (**a**), TiDPPSH (**b**), VDPPSH (**c**), CrDPPSH (**d**), MnDPPSH (**e**), FeDPPSH (**f**), CoDPPSH (**g**), CuDPPSH (**h**) systems, respectively. In the plots, the green isosurfaces represent the positive phase of wave function, and the red isosurfaces represent the negative phase of wave function of the molecular orbitals.

Figure 3 provides the molecular orbital electron distribution of the highest occupied molecular orbital (HOMO) and lowest unoccupied molecular orbital (LUMO) for thiol-terminated MPs. The electronic structure, specifically the eigenvalues associated with the HOMO and LUMO, along with their energy gaps, and identifies the chemical reactivity of molecules [23]. In each of the MDPPSH systems, the HOMO and LUMO are primarily located on the metalloporphyrin plane. However, the d-orbitals of the central metal atoms exhibit varying degrees of overlap with the porphyrin orbitals, likely due to the different spin multiplicities of the metal centers. ScDPPSH has an unpaired electron, resulting in a doublet spin multiplicity ground state, exhibiting d-p orbital interaction, leading to electron density being delocalized around the Sc and N atoms. Unlike ScDPPSH, TiDPPSH has two unpaired electrons, resulting in a triplet spin multiplicity. Despite this difference, it also shows d-p interaction. The VDPPSH and MnDPPSH systems with unpaired electrons (high-spin state) show electron density more localized around the d-orbitals of central metals. Despite its high-spin state, CrDPPSH has no localized electron density on Cr. Here, we find that the spin state of the atom remains the same even at the center of the macrocycle, meaning that there is no quenching of the spin state.

In FeDPPSH, CoDPPSH, and CuDPPSH systems, the HOMOs show predominant electron delocalization on the nitrogen and carbon atoms within the porphyrin plane. Conversely, the LUMOs (antibonding orbitals) of all MDPPSH systems are situated primarily within the metalloporphyrin core with d-p interactions and exhibit minimal variation compared to the HOMOs. This suggests metal–porphyrin interaction within the core for all MDPPSH systems, with electron transfer from the metals to the porphyrin. Interestingly, the -SH anchoring group in Cr, Fe, Co, and Cu appears to impact the porphyrin core electron density in the HOMOs. The combined effect of the -SH and phenyl groups likely depletes electron density on the respective metals. We observed a similar effect in the case of MDPPSeH, as shown in Figure S3. In contrast, the -TeH anchoring groups exhibit insignificantly trivial HOMO and LUMO electron density in the studied MDPPTeH molecules, distinguishing them from the -SH and -SeH anchoring groups (see Figure S4). The details of the HOMO and LUMO electronic structures for MDPPSeH and MDPPTeH are explained in the Supporting Information (see Figures S3 and S4 and Section 1.2). In summary, MPP interactions, spin states, and the influence of functional groups play pivotal roles in shaping the electronic structure and reactivity of MDPPSH, MDPPSeH, and MDPPTeH systems.

**Figure 4:**
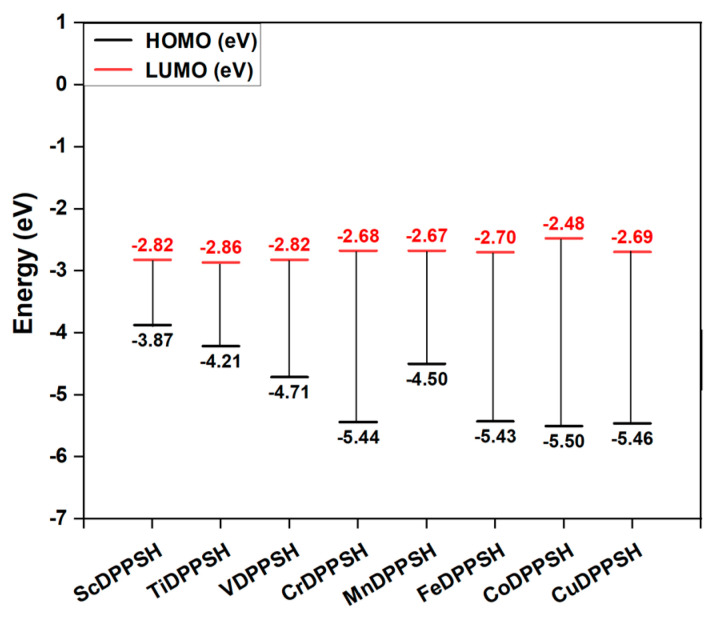
The HOMO and LUMO for MDPPSH (M = Sc--Cu) (Data in Figure 4 refer to global HOMO and global LUMO orbitals).

Figure 4 reveals a trend of increasingly negative HOMO and LUMO energies across MDPPSH systems (M = Sc--Cu), suggesting generally lower overall energy levels for the molecular orbitals. This could be attributed to the increasing number of electrons filling the 3d-orbitals in the metals as we move from lighter (Sc) to heavier (Cu) elements. HOMO energies range from −3.87 eV (ScDPPSH, highest) to −5.50 eV (CoDPPSH, lowest), indicating easier electron removal (lower ionization energy) for CoDPPSH. LUMO energies show a less pronounced trend (−2.48 to −2.86 eV), with CoDPPSH having the highest value but not significantly different from others. This suggests that the ability of these MDPPSH molecules to accept electrons (electron affinity) might not vary as drastically as the HOMO energies. Overall, CoDPPSH exhibits the most negative HOMO and LUMO energy levels, stretching the gap between them compared to other MDPPSH systems. It is important to remember that within each HOMO and LUMO level of MDPPSH, there are two spin states for the electron, spin-up, and spin-down (See Table S6 of Supporting Information). For Sc to Cr in MDPPSH, the spin-up HOMO contributes to the global HOMO energy level, while for Mn to Cu, the spin-down HOMO dictates the global HOMO. Similarly, the global LUMO comes from spin-down for most MDPPSH systems (except VDPPSH and CoDPPSH) (See Table S6 of Supporting Information). The HOMO and LUMO energy levels for -SeH and -TeH are detailed in the Supporting Information (Figure S5). Overall, CoDPPSeH and CoDPPTeH exhibit the highest HOMO and LUMO energies, resulting in the largest gaps between them compared to other MDPPSeH and MDPPTeH systems. There is no notable change in the electronic structure among the -SH, -SeH, and -TeH groups, as they behave similarly (Tables S8 and S9 of Supporting Information).

All MDPPSH (M = Sc--Cu) molecules exhibit semiconducting behavior with 1.05 to 3.03 eV energy gaps (Table S7 of Supporting Information). This variation originates from metal-dependent orbital deformations (see Figure 3). These deformations alter the conjugation within the metalloporphyrin rings and shift HOMO and LUMO energy levels and the observed range of energy gaps [24]. ScDPPSH exhibits the smallest gap (1.05 eV), suggesting it might be a better conductor and could potentially absorb longer wavelength light compared to CoDPPSH, which has the largest gap (3.03 eV). This lower energy barrier could also facilitate efficient electron transfer in applications like dye-sensitized solar cells [25]. Delving deeper, we can explore the influence of spin states within each HOMO and LUMO level (Table S6 of Supporting Information). These levels can accommodate electrons with two spin orientations (up and down). Interestingly, MDPPSH systems with later 3d-series metals (Cr, Fe, Cu) show minimal differences between spin-up and spin-down HOMO-LUMO gaps. Notably, CoDPPSH is the only case where both spin states have identical energy gaps, suggesting a more uniform electronic structure. Conversely, MDPPSH systems with earlier 3d-series metals (Sc, Ti, V, Mn) exhibit a more pronounced difference between spin-up and spin-down gaps (Table S6 of Supporting Information). For instance, ScDPPSH, TiDPPSH, VDPPSH, and MnDPPSH showed changes above 29%. This highlights the significant role of spin states in shaping the overall energy landscape for these materials. Since the spin state remains consistent across MDPPSH, MDPPSeH, and MDPPTeH systems (for details, see Tables S10 and S11 in Section 1.2 of Supporting Information), the electronic behavior in terms of spin-up and spin-down remains similar to that of MDPPSH. This finding underscores that the choice of anchoring group -SH, -SeH, or -TeH does not significantly alter the spin properties of these first-row 3d-MDPP systems. The observed difference in spin gaps caused by the functionalization of d-orbitals suggests potential applications in spintronic devices. These devices rely on manipulating the spin state of electrons, and the turn-on voltage threshold depends heavily on the choice of central transition metal (Sc--Cu) and its functional group [26].

As shown in Table 2, Tables S12 and S13, the charge on the central metal generally decreases from Sc to Cu across all systems, reflecting the different electron affinities and bonding characteristics of these metals. The nitrogen atoms in the porphyrin ring consistently accumulate small positive charges across all systems, playing a role in stabilizing the metal-porphyrin core. ScDPPSH/SeH/TeH shows the highest charge on the central metal with the least charge on C2_pp_, indicating the Sc metal atom has strong electron retention and minimal interaction with C2_pp_. CoDPPSH exhibits high charges on both the metal and C2_pp_, suggesting strong electron interactions and retention. FeDPPSH and CuDPPSH also show high charge accumulation on C2_pp_, indicating significant electronic influence from the central metal. The SH group shows significant electron density depletion, while SeH shows minimal depletion and TeH exhibits electron density accumulation. This phenomenon arises due to the lower electronegativity of TeH compared to SH and SeH anchoring groups. Thus, the charge distribution variations are logical outcomes influenced by both the central metal atom and the specific anchoring group within the MDPPSH/SeH/TeH systems.

### 2.4. Conceptual DFT-Based Electronic Structure Reactivity Descriptors for Thiol-Terminated Metalloporphyrins

DFT-based electronic structure reactivity descriptors, µ, η, σ, and ω were analyzed to discern how they vary with different central metals in MDPPSH systems as shown in Table 3. The electronic chemical potential (µ, as per Equation (S1)) reflects the propensity of a molecule to gain or lose electrons. ScDPPSH exhibited the highest electronic chemical potential (−3.35 eV), while CuDPPSH displayed the lowest (−4.08 eV). Global hardness (η, as per Equation (S2)) represents the resistance of a molecule to the deformation of its electron cloud. CoDPPSH displayed the highest η value (3.03 eV), indicating greater stability, whereas ScDPPSH exhibited the lowest (1.05 eV), suggesting lower stability. Conversely, global softness (σ, as per Equation (S3)) is inversely related to global hardness, with higher values indicating a greater tendency for the electron cloud to deform and participate in reactions. ScDPPSH showed the highest σ value, suggesting it is softer and more polarizable than other systems. The electrophilicity index (ω, as per Equation (S4)) estimates the ability of a molecule to accept electrons, with higher values indicating greater electrophilicity. ScDPPSH demonstrated the highest ω value (5.33 eV), indicating it is more electrophilic compared to others. Overall, among the MDPPSH systems studied, CoDPPSH exhibited a higher band gap, η values, and a lower value of ω, which shows high stability and the least reactivity compared to others.

### 2.5. Comparison of Thiol-Terminated Metalloporphryins to -SeH- and -TeH-Terminated Metalloporphyrins

The ground state molecular conformations of MDPPSeH and MDPPTeH (Sc--Cu) are shown in Figures S1 and S2 and geometric data can be found in Tables S4 and S5, respectively. The macrocycle metal-based porphyrins with -SeH and -TeH show no apparent distortion and one may consider it as “perfectly flat” similar to the -SH-terminated molecules. These similarities continue as the trend seen for variation in the C-C covalent bond lengths as one moves from Sc to Cu remains no matter the terminal group.

The electron density distributions for MDPPSeH and MDPPTeH are in Figures S3 and S4, respectively. Notably, the HOMO orbitals display delocalization across the metal atoms (Sc-V, Mn) and nitrogen atoms, with the exception of Cr, Fe, Co, and Cu for MDPPSeH. However, the d-orbitals of the central metal atoms exhibit varying degrees of overlap with the porphyrin orbitals, likely due to the different spin multiplicities of the metal centers. V, Mn, and Cr-MDPP are high-spin systems among other systems. Conversely, the LUMO orbitals (Figure S3), representing antibonding orbitals, are predominantly situated in the center of the metalloporphyrin plane with d-p interactions. We found that this was observed in -SH and -TeH porphyrins in this study.

The spin state of the 3d TMs remains consistent across MDPPSH, MDPPSeH, and MDPPTeH, indicating that the choice of -SH, -SeH, or -TeH anchoring groups does not significantly affect the spin state of central TMs in MP systems. Like MDPPSH systems, later 3d-series metals (Cr, Fe, Cu) show minimal differences between spin-up and spin-down HOMO-LUMO gaps in MDPPSeH and MDPPTeH systems. Notably, CoDPPSeH and CoDPPTeH are the only cases where both spin states have identical energy gaps, suggesting a more uniform electronic structure. Conversely, MDPPSeH and MDPPTeH systems with earlier 3d-series metals (Sc, Ti, V, Mn) exhibit a more pronounced difference between spin-up and spin-down gaps (Tables S6, S7, S10 and S11).

However, a notable distinction arises in the Mulliken charges associated with these anchoring groups. Specifically, both -SH and -SeH groups exhibit negative Mulliken charges (Tables S12 and S13), whereas the -TeH group displays a positive Mulliken charge. This difference can be attributed to Te’s lower electronegativity compared to S and Se, resulting in a weaker electron-withdrawing effect for -TeH.

This trend is further supported by the electrostatic potential (ESP) maps, which show strongly negative potential (blue regions) near the S and Se atoms, but neutral to slightly positive potential (green-yellow regions) around the Te atoms (Figures S6–S8). These ESP surfaces visually reinforce the Mulliken population analysis, confirming reduced electron localization at the Te sites, consistent with increasing atomic radius and polarizability along the chalcogen series. Additionally, the ESP maps reveal systematic variations near the metal center across the 3d transition series. Early transition metals (Sc, Ti) exhibit diffuse, symmetric positive potentials, reflecting lower d-electron density. Mid-series metals (Cr, Mn) display strongly polarized and directional ESP features, consistent with localized spin density in high-spin configurations. In contrast, late transition metals (Fe, Co, Cu) show more delocalized and smoother ESP distributions, indicating enhanced metal-ligand back-donation and greater electronic stabilization. These observations are in excellent agreement with the Mulliken charge distributions (Table 2) and spin multiplicity data (Table S1), confirming that the electron distribution in these systems is governed by both the d-electron configuration of the central metal and the electronegativity and polarizability of the terminal -XH (X = S, Se, Te) ligands.

The details about DFT-based electronic structure reactivity descriptors for MDPPSeH and MDPPTeH can be found in Tables S14 and S15. Of note, in all cases, the ScDPPXH and CuDPPXH (X = S, Se, and Te) displayed the highest and lowest chemical potential. These trends held for η values, ω, and σ, meaning the same molecules maintained the same trends across the series of S, Se, and Te groups.

## 3. Discussion

Our analysis focused on geometric configurations, orbital energies, spin multiplicity, and reactivity descriptors to understand the impact of different central 3d-metals by changing the anchoring groups from -SH to -SeH and -TeH. These changes have little implication on the spin multiplicity of the molecule; however, they do not negate the possible role of these groups for molecular junctions. For example, Yang and co-workers showed that the rotation of single-molecule devices causes spin switching in part due to a change in electron distribution [27]. This phenomenon could be at play in molecules with phenyl groups given the ability to rotate. In addition, we observed slight decreases in the HOMO/LUMO energies in MDPP systems with -TeH compared to those with -SH and -SeH across all studied metals (M = Sc--Cu). This change may alter the molecular orbital energy alignment with the gold electrode bands between the junction, which is important in spintronic applications [6].

The DFT-based global chemical reactivity descriptor shows that among all MDPP systems (M = Sc--Cu), CoDPPSH/SeH/TeH, those with higher band gaps, higher η values, and a lower value of σ and ω are more stable and less reactive compared to others in this study. This finding correlates with the wide use of cobalt porphyrins over the years for studies in catalysis, sensing, and molecular electronics.

Considering these findings, replacing -SH with -SeH or -TeH may offer advantages in terms beyond just the transport properties of metalloporphyrins in molecular electronic devices. Additionally, this work provides valuable insight on the use of chalcogens beyond sulfur as an anchoring group towards optimizing the performance of metalloporphyrins in technological applications.

## 4. Materials and Methods

The calculations utilized a recent quantum chemistry program Gaussian16 Rev. C.02. The Becke three-parameter hybrid functional (B3LYP) method with Becke–Johnson damping for dispersion correction offered a good balance of accuracy and efficiency for transition metals [28,29,30]. For metals and non-metal atoms, a combination of Def2SVP/6-311+G* basis sets was employed to improve electronic and bonding descriptions [31,32]. SCF convergence was achieved with energy changes between iterations smaller than 10^−6^ Hartree, and density matrix convergence thresholds of 10^−8^ RMS and 10^−6^ maximum were applied. Geometry optimization convergence criteria were set to a maximum force of 4.5 × 10^−4^ Hartree/Bohr, RMS force of 3.0 × 10^−4^ Hartree/Bohr, maximum displacement of 0.0018 Å, and RMS displacement of 0.0012 Å, all of which were satisfied in the optimization process.

Initial optimizations were performed, starting from symmetric structures and iteratively explored multiple spin states until both energy and geometry converged, ensuring that the most stable electronic configuration was identified (see Table S1). In all cases, the optimized geometries converged to C₁ point group symmetry, regardless of spin multiplicity. This low symmetry results from the steric and electronic effects of the meso-substituted phenyl rings and the terminal -XH (X = S, Se, Te) anchoring groups, which collectively distort the idealized planarity of the porphyrin macrocycle. The fact that this asymmetry persisted across spin states further confirms that it originates from substituent-induced structural effects, rather than spin state-dependent distortions. Frequency calculations were performed for all optimized geometries to verify their stability. These calculations confirmed that each structure corresponds to a true minimum on the potential energy surface.

Beyond a single parameter, stability analysis delved into specific bond lengths, angles, and vibrational frequencies. Frontier orbital energies (HOMO and LUMO) were calculated using the B3LYP-D3 method to understand orbital interactions and potential reactivity. Additional DFT-derived descriptors like electronic chemical potential, hardness, softness, and electrophilicity index were calculated based on referenced equations, providing further insights into reactivity and electron transfer [33,34,35]. The electronic chemical potential (μ) represents a system propensity to gain or lose electrons, signifying its electron escaping tendency at equilibrium. Global hardness (η) describes the system resistance to charge transfer, whereas global softness (σ), its reciprocal, characterizes the system polarizability and ease of charge redistribution. The electrophilicity index (ω) quantifies a system ability to accept electrons, thereby assessing its electrophilic nature and reactivity toward electron-rich species. More details of electronic chemical potential, hardness, softness, and electrophilicity index are provided in Supporting Information and our previous reports [17].

## 5. Conclusions

Using DFT, we have illustrated that changing the chalcogen anchoring group may offer advantages in terms beyond just the transport properties of metalloporphyrins in molecular electronic devices. The changes in electronic structure, though subtle, reveal that -TeH may not only play a role in transport properties, but may increase chemical hardness, that is stability. We hope this study encourages other researchers to explore other chalcogen anchoring groups and explore future simulations employing non-equilibrium Green’s functions with MDPPTeH between gold electrodes.

## Figures and Tables

**Table 1:** The geometrical parameters bond length (Å) and dihedral angle lengths ( ° ) of MDPPSH systems.

System	M-N	N-C1_pp_	C1_pp_-C_β_	C_β_-C_β′_	C1_pp_-C2_pp_-C3_Ph_-C4_Ph_
ScDPPSH	2.09	1.38	1.43	1.37	69.23
TiDPPSH	2.07	1.39	1.44	1.37	67.93
VDPPSH	2.06	1.38	1.43	1.36	68.26
CrDPPSH	2.05	1.38	1.44	1.36	69.09
MnDPPSH	2.03	1.38	1.44	1.36	69.64
FeDPPSH	2.01	1.38	1.44	1.36	70.63
CoDPPSH	1.99	1.37	1.43	1.36	71.29
CuDPPSH	2.03	1.37	1.44	1.36	69.97

**Table 2:** Mulliken population distribution for MDPPSH (M = Sc--Cu) systems.

System	M	N	C2_pp_	SH
ScDPPSH	0.99	0.01	0.06	−0.50
TiDPPSH	0.50	0.20	0.08	−0.50
VDPPSH	0.58	0.22	0.20	−0.50
CrDPPSH	0.53	0.26	0.29	−0.50
MnDPPSH	0.70	0.25	0.24	−0.50
FeDPPSH	0.76	0.26	0.61	−0.50
CoDPPSH	0.87	0.32	0.63	−0.50
CuDPPSH	0.60	0.27	0.61	−0.50

**Table 3:** The calculated values of electronic chemical potential (µ), global hardness (η), global softness (σ), and electrophilicity index (ω) for MDPPSH (M = Sc--Cu), respectively.

System	µ (eV)	η (eV)	σ (eV)	ω (eV)
ScDPPSH	−3.35	1.05	0.48	5.33
TiDPPSH	−3.54	1.35	0.37	4.64
VDPPSH	−3.77	1.89	0.26	3.75
CrDPPSH	−4.06	2.76	0.18	2.98
MnDPPSH	−3.59	1.82	0.27	3.53
FeDPPSH	−4.06	2.73	0.18	30.3
CoDPPSH	−3.99	3.03	0.17	2.63
CuDPPSH	−4.08	2.76	0.18	3.01

## Data Availability

The original contributions presented in this study are included in the article/Supplementary Materials. Further inquiries can be directed to the corresponding author.

## Supplementary Materials

The following supporting information can be downloaded at https://www.mdpi.com/article/10.3390/molecules30112254/s1, Figures S1 and S2: Optimized structures of metals-based diphenyl porphyrin with selenium and tellurium; Figures S3 and S4: Spatial distributions of electron density for MDPPSeH and MDPPTeH; Figure S5: The global energies of HOMO and LUMO are represented for MDPPSeH and MDDPPTeH (M = Sc--Cu); Figures S6–S8: The electrostatic potential maps for MDPPSH/MDPPSeH/MDPPTeH; Table S1: Spin multiplicity of all systems studied; Table S2: Relative ground state energies for selected MPPSH species; Table S3: Comparisons of bond lengths of MDPPSH to previous reports; Tables S4 and S5: Geometrical parameters of MDPPSeH/MDPPTeH; Tables S6: HOMO-LUMO energy data for MDPPSH/MDPPSeH/MDPPTeH systems; Tables S12 and S13: Mulliken population distribution for MDPPSeH/MDPPTeH; Tables S14 and S15: Conceptual DFT-based reactivity descriptors for MDPPSeH/MDPPTeH. References [17,33,34,35,36,37,38,39,40,41,42,43] are cited in the Supplementary Materials.

## Abbreviations

The following abbreviations are used in this manuscript:

DFT	Density Functional Theory
