# Supplementary material for: Exploring the Influence of Chalcogens on Metalloporphyrins: A DFT Study
Source: Molecules. 2025 May 22;30(11):2254. doi: 10.3390/molecules30112254 (PMC12155939; doi:10.3390/molecules30112254)
Supplement: Supplementary file 1 [file molecules-30-02254-s001.zip › molecules-3615863-supplementary.pdf]

Supporting Information

Exploring the Influence of Chalcogens on Metalloporphyrins: A DFT Study

Beenish Bashir [1] and Andre Z. Clayborne [1,2,*]

1 Department of Chemistry and Biochemistry, George Mason University, Fairfax, VA 22030, USA;
2 Quantum Science and Engineering Center, George Mason University, Fairfax, VA 22030, USA
* Correspondence: aclaybo@gmu.edu

1. Supplemental Data

DFT-derived descriptors

DFT-derived descriptors like electronic chemical potential (equation S1), hardness (equation S2), softness (equation S3), and electrophilicity index (equation S4) were calculated based on referenced equations, providing further insights into reactivity and electron transfer.[1-4]

$$\mu \simeq -\frac{1}{2}\left(E_{HOMO} + E_{LUMO}\right) \tag{S1}$$

$$\eta \simeq \left(E_{LUMO} - E_{HOMO}\right) \tag{S2}$$

$$\sigma \simeq \frac{1}{2\eta} \tag{S3}$$

$$\omega \simeq \frac{\mu^2}{2\eta} \tag{S4}$$

Table S1. The spin multiplicity values of all MDPPX (M = Sc--Cu) systems with (X = -SH, -SeH, and -TeH) anchoring groups.

Systems	-SH	-SeH	-TeH
ScDPP	2	2	2
TiDPP	3	3	3
VDPP	4	4	4
CrDPP	5	5	5
MnDPP	4	4	4
FeDPP	3	3	3
CoDPP	2	2	2
CuDPP	2	2	2

Table S2. Relative ground state energies (in eV) of VDPPSH, CrDPPSH, and MnDPPSH for various spin multiplicities, with respect to the lowest energy state.

Systems	Spin Multiplicity	Relative Energy (eV)
VDPPSH	2	−0.594
	4	0.0
	6	1.495
CrDPPSH	1	3.463
	3	1.174

	5	0.0
MnDPPSH	2	0.949
	4	0.170
	6	0.0

Table S3. The comparison of M-N bond length (Å) of different MDPPSH (M = Ti, Cr, Mn, Fe, Co, and Cu) with the literature reported.

Systems	Current work (M-N) Å	Literature (M-N) Å
TiDPPSH	2.074	~ 2.085 [5]
CrDPPSH	2.049	2.033 [6]
MnDPPSH	2.027	~ 2.073 [7]
FeDPPSH	2.007	~ 2.004 [7-8]
CoDPPSH	1.994	1.985 [8-9]
CuDPPSH	2.027	~ 1.991 [10-11]

1.1. Ground state structures and structural analysis metals-based diphenyl porphyrin with selenium and Tellurium (MDPPSeH/TeH; M=Sc-Cu)

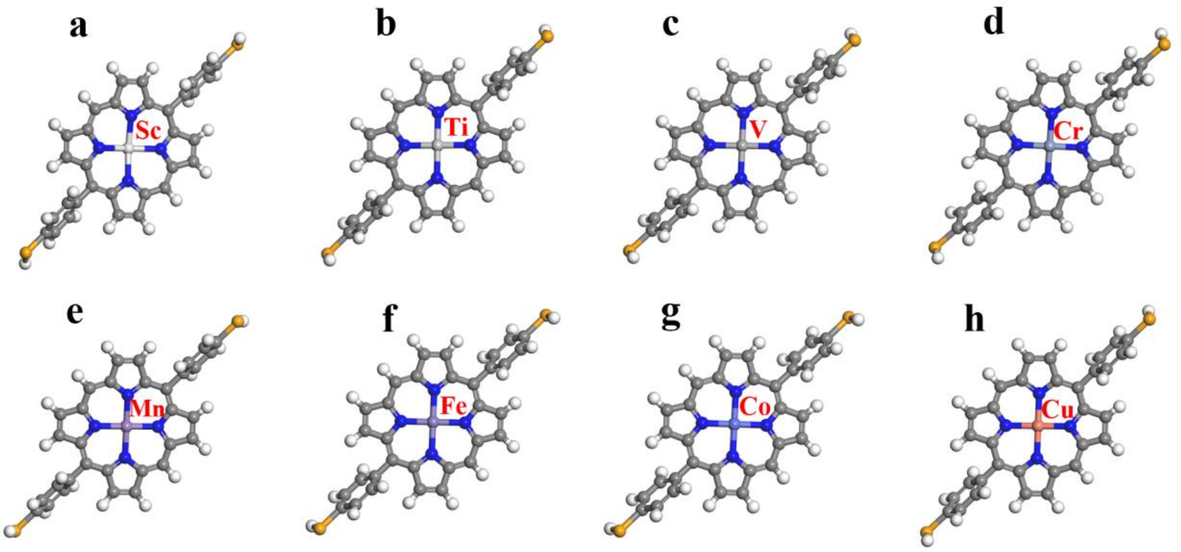

Figure S1. Optimized structures of metals-based diphenyl porphyrin with selenium (ScDPPSeH (a), TiDPPSeH (b), VDPPSeH (c), CrDPPSeH (d), MnDPPSeH (e), FeDPPSeH (f), CoDPPSeH (g), CuDPPSeH (h) systems, respectively.

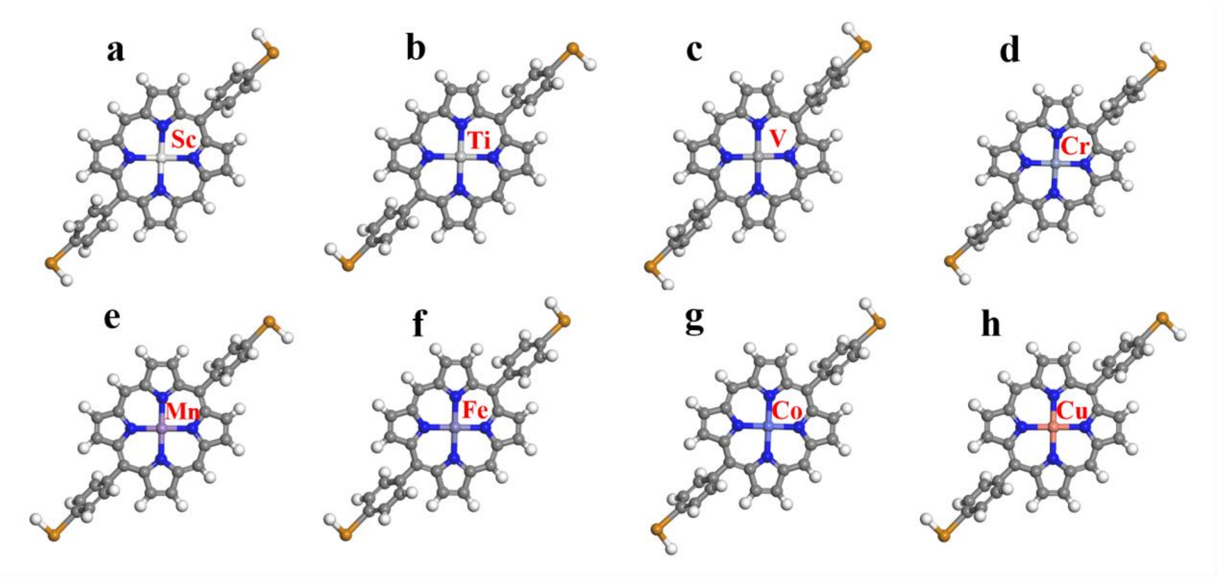

Figure S2. Optimized structures of metals-based diphenyl porphyrin with tellurium (ScDPPTeH (a), TiDPPTeH (b), VDPPTeH (c), CrDPPTeH (d), MnDPPTeH (e), FeDPPTeH (f), CoDPPTeH (g), CuDPPTeH (h) systems, respectively).

Table S4. The geometrical parameters bond length (Å), and dihedral angles (˚) of MDPPSeH systems (M = Sc--Cu).

System	M-N	N-C1$_{pp}$	C1$_{pp}$-C$_{\beta}$	C$_{\beta}$-C$_{\beta'}$	C1$_{pp}$- C2$_{pp}$-C3$_{Ph}$-C4$_{Ph}$
ScDPPSeH	2.11	1.39	1.43	1.37	69.58
TiDPPSeH	2.05	1.38	1.44	1.37	68.29
VDPPSeH	2.06	1.38	1.44	1.36	68.59
CrDPPSeH	2.05	1.38	1.44	1.36	69.40
MnDPPSeH	2.03	1.38	1.44	1.36	69.93
FeDPPSeH	2.01	1.38	1.44	1.36	70.95
CoDPPSeH	1.99	1.37	1.44	1.36	71.60
CuDPPSeH	2.03	1.37	1.44	1.36	70.03

Table S5. The geometrical parameters bond length, and dihedral angles of MDPPTeH systems (M = Sc--Cu).

System	M-N	N-C1$_{pp}$	C1$_{pp}$-C$_{\beta}$	C$_{\beta}$-C$_{\beta'}$	C1$_{pp}$- C2$_{pp}$-C3$_{Ph}$-C4$_{Ph}$
ScDPPTeH	2.09	1.39	1.43	1.37	69.31
TiDPPTeH	2.07	1.38	1.44	1.37	68.35
VDPPTeH	2.06	1.38	1.44	1.36	68.75
CrDPPTeH	2.05	1.38	1.44	1.36	70.28
MnDPPTeH	2.03	1.38	1.44	1.36	70.13
FeDPPTeH	2.01	1.38	1.44	1.36	71.82
CoDPPTeH	1.99	1.37	1.44	1.36	71.99
CuDPPTeH	2.03	1.38	1.44	1.36	70.41

1.2. Electronic Properties of MDPPSeH and MDPPTeH (M = Sc--Cu)

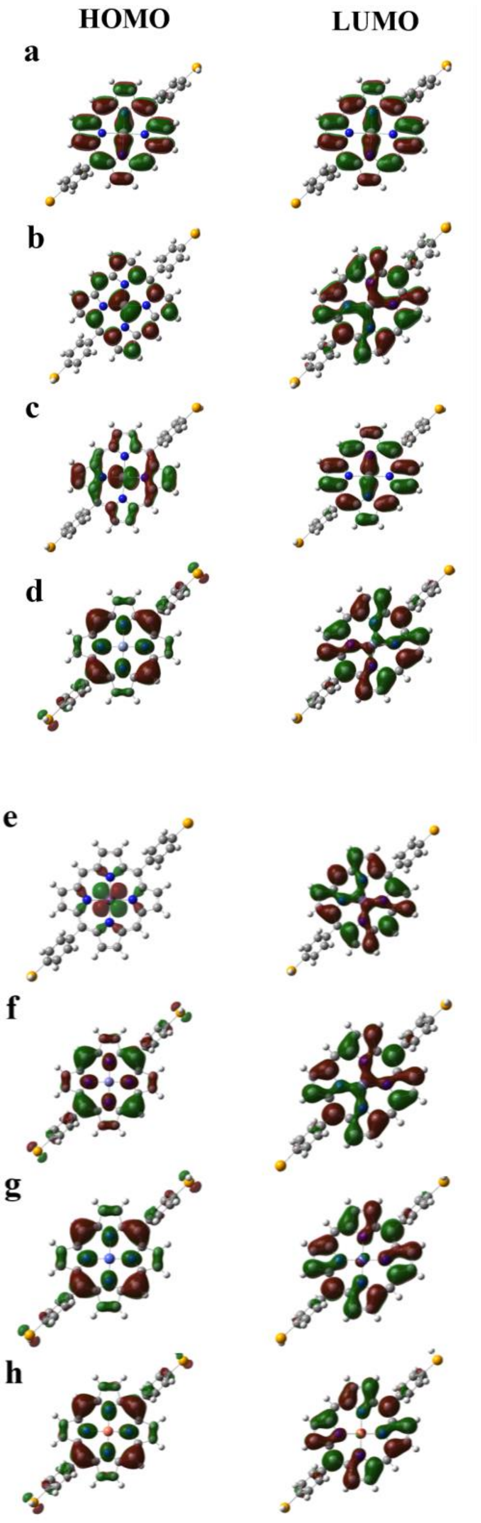

Figure S3. Spatial distributions of HOMO and LUMO calculated for ScDPPSeH (a), TiDPPSeH (b), VDPPSeH (c), CrDPPSeH (d), MnDPPSeH (e), FeDPPSeH (f), CoDPPSeH (g), CuDPPSeH (h) systems, respectively.

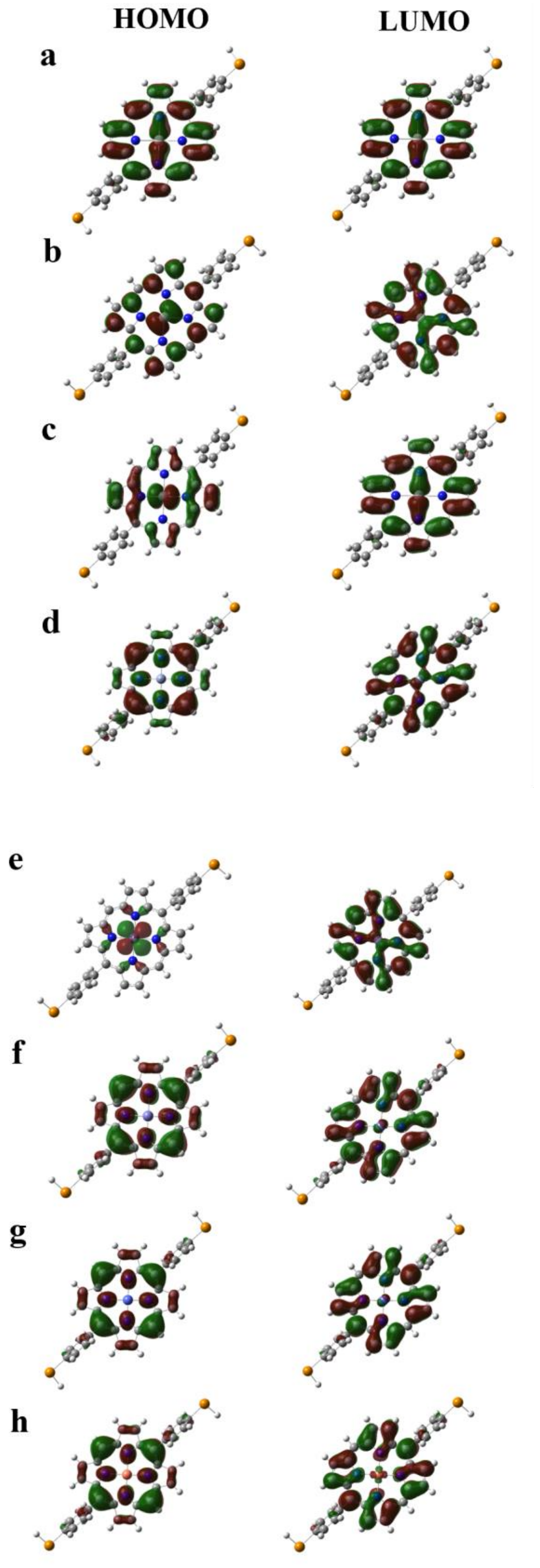

Figure S4. Spatial distributions of HOMO and LUMO calculated for ScDPPTeH (a), TiDPPTeH (b), VDPPTeH (c), CrDPPTeH (d), MnDPPTeH (e), FeDPPTeH (f), CoDPPTeH (g), CuDPPTeH (h) systems, respectively.

Table S6. The energies of HOMO and LUMO for spin-up and spin-down are represented for MDPPSH (M = Sc--Cu) systems.

Systems	Spin	HOMO (eV)	LUMO (eV)	Gap (eV)

ScDPPSH	Up	−3.87	−2.69	1.18
	Down	−5.33	−2.82	2.51
TiDPPSH	Up	−4.21	−2.80	1.4
	Down	−5.43	−2.86	2.56
VDPPSH	Up	−4.71	−2.82	1.89
	Down	−5.43	−2.74	2.69
CrDPPSH	Up	−5.44	−2.49	2.95
	Down	−5.46	−2.68	2.79
MnDPPSH	Up	−5.43	−2.49	2.94
	Down	−4.5	−2.67	1.82
FeDPPSH	Up	−5.47	−2.52	2.96
	Down	−5.43	−2.7	2.73
CoDPPSH	Up	−5.51	−2.48	3.03
	Down	−5.50	−2.47	3.03
CuDPPSH	Up	−5.49	−2.56	2.94
	Down	−5.46	−2.69	2.76

Table S7. HOMO-LUMO gaps of MDPPSH (M = Sc--Cu) systems.

Systems	HOMO-LUMO Gap (eV)
ScDPPSH	1.05
TiDPPSH	1.35
VDPPSH	1.89
CrDPPSH	2.76
MnDPPSH	1.82
FeDPPSH	2.73
CoDPPSH	3.03
CuDPPSH	2.76

Table S8. HOMO-LUMO gaps of MDPPSeH (M = Sc--Cu) systems.

Systems	HOMO-LUMO Gap (eV)
ScDPPSeH	1.05
TiDPPSeH	1.35
VDPPSeH	1.89
CrDPPSeH	2.76
MnDPPSeH	1.82
FeDPPSeH	2.72
CoDPPSeH	3.03
CuDPPSeH	2.77

Table S9. HOMO-LUMO gaps of MDPPTeH (M = Sc--Cu) systems.

Systems	HOMO-LUMO Gap (eV)
ScDPPTeH	1.05
TiDPPTeH	1.34
VDPPTeH	1.89
CrDPPTeH	2.78
MnDPPTeH	1.82
FeDPPTeH	2.68
CoDPPTeH	3.05
CuDPPTeH	2.80

Table S10. The energies of HOMO and LUMO for spin-up and spin-down are represented for MDPPSeH (M = Sc--Cu) systems.

Systems	Spin	HOMO (eV)	LUMO (eV)	Gap (eV)

ScDPPSeH	Up	−3.90	−2.72	1.18
	Down	−5.37	−2.85	2.52
TiDPPSeH	Up	−4.24	−2.84	1.40
	Down	−5.45	−2.89	2.57
VDPPSeH	Up	−4.73	−2.84	1.89
	Down	−5.46	−2.77	2.69
CrDPPSeH	Up	−5.46	−2.51	2.95
	Down	−5.49	−2.70	2.79
MnDPPSeH	Up	−5.46	−2.51	2.94
	Down	−4.52	−2.70	1.82
FeDPPSeH	Up	−5.50	−2.54	2.96
	Down	−5.45	−2.72	2.72
CoDPPSeH	Up	−5.53	−2.50	3.03
	Down	−5.52	−2.49	3.03
CuDPPSeH	Up	−5.52	−2.58	2.94
	Down	−5.49	−2.72	2.77

Table S11. The energies of HOMO and LUMO for spin-up and spin-down are represented for MDPPTeH (M = Sc--Cu) systems.

Systems	Spin	HOMO (eV)	LUMO (eV)	Gap (eV)
ScDPPTeH	Up	−3.95	−2.76	1.18
	Down	−5.44	−2.90	2.54
TiDPPTeH	Up	−4.27	−2.88	1.39
	Down	−5.55	−2.94	2.61
VDPPTeH	Up	−4.78	−2.89	1.89
	Down	−5.54	−2.82	2.72
CrDPPTeH	Up	−5.54	−2.57	2.97
	Down	−5.57	−2.76	2.81
MnDPPTeH	Up	−5.53	−2.57	2.97
	Down	−4.57	−2.75	1.82
FeDPPTeH	Up	−5.58	−2.57	3.02
	Down	−5.44	−2.76	2.68
CoDPPTeH	Up	−5.60	−2.55	3.05
	Down	−5.59	−2.54	3.05
CuDPPTeH	Up	−5.60	−2.63	2.96
	Down	−5.56	−2.76	2.80

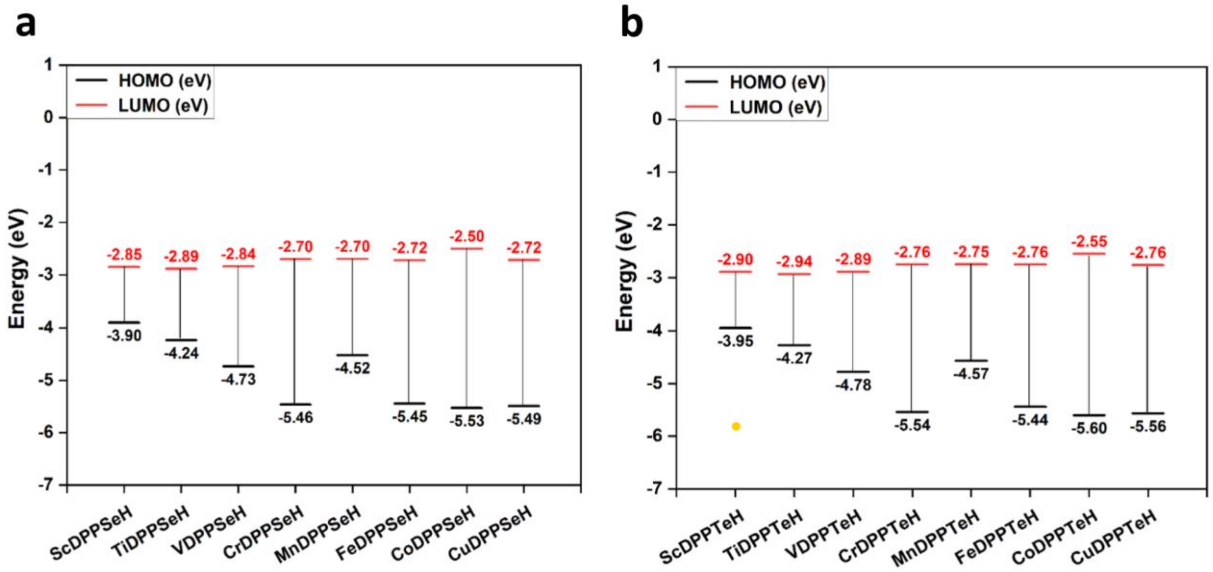

Figure S5. The global energies of HOMO and LUMO are represented for MDPPSeH and MDDPPTeH (M = Sc--Cu).

Table S12. Mulliken population distribution for MDPPSeH (M = Sc--Cu) systems.

System	M	N	C2pp	SeH
ScDPPSeH	0.98	0.01	0.05	-0.06
TiDPPSeH	0.57	0.20	0.08	-0.06
VDPPSeH	0.59	0.22	0.21	-0.06
CrDPPSeH	0.58	0.26	0.23	-0.06
MnDPPSeH	0.70	0.25	0.13	-0.06
FeDPPSeH	0.75	0.26	0.42	-0.06
CoDPPSeH	0.86	0.31	0.64	-0.06
CuDPPSeH	0.55	0.28	0.59	-0.06

Table S13. Mulliken population distribution for MDPPTeH (M = Sc--Cu) systems.

System	M	N	C2pp	TeH
ScDPPTeH	1.00	0.01	0.02	0.21
TiDPPTeH	0.54	0.19	0.04	0.21
VDPPTeH	0.59	0.21	0.23	0.21
CrDPPTeH	0.53	0.27	0.24	0.21
MnDPPTeH	0.72	0.26	0.15	0.21
FeDPPTeH	0.80	0.30	0.59	0.21
CoDPPTeH	0.85	0.31	0.65	0.21
CuDPPTeH	0.61	0.29	0.61	0.21

1.3. Conceptual DFT-based global reactivity descriptors for MDPPSeH/TeH (M= Sc--Cu)

Table S14. The electronic chemical potential (μ), global hardness (η), global softness (σ), and electrophilicity index (ω) are calculated for MDPPSeH (M= Sc--Cu).

System	μ (eV)	η (eV)	σ (eV)	ω (eV)
ScDPPSeH	−3.37	1.05	0.48	5.42
TiDPPSeH	−3.56	1.35	0.37	4.70
VDPPSeH	−3.79	1.89	0.26	3.79
CrDPPSeH	−4.08	2.76	0.18	3.02
MnDPPSeH	−3.61	1.82	0.27	3.58

FeDPPSeH	−4.09	2.72	0.18	3.06
CoDPPSeH	−4.01	3.03	0.17	2.66
CuDPPSeH	−4.10	2.77	0.18	3.04

Table S15. The electronic chemical potential (μ), global hardness (η), global softness (σ), and electrophilicity index (ω) are calculated for MDPPTeH (M = Sc--Cu).

System	μ (eV)	η (eV)	σ (eV)	ω (eV)
ScDPPTeH	−3.42	1.05	0.48	5.57
TiDPPTeH	−3.60	1.35	0.37	4.86
VDPPTeH	−3.84	1.89	0.26	3.90
CrDPPTeH	−4.15	2.78	0.18	3.09
MnDPPTeH	−3.66	1.82	0.28	3.68
FeDPPTeH	−4.10	2.68	0.19	3.13
CoDPPTeH	−4.08	3.05	0.16	2.73
CuDPPTeH	−4.16	2.80	0.18	3.10

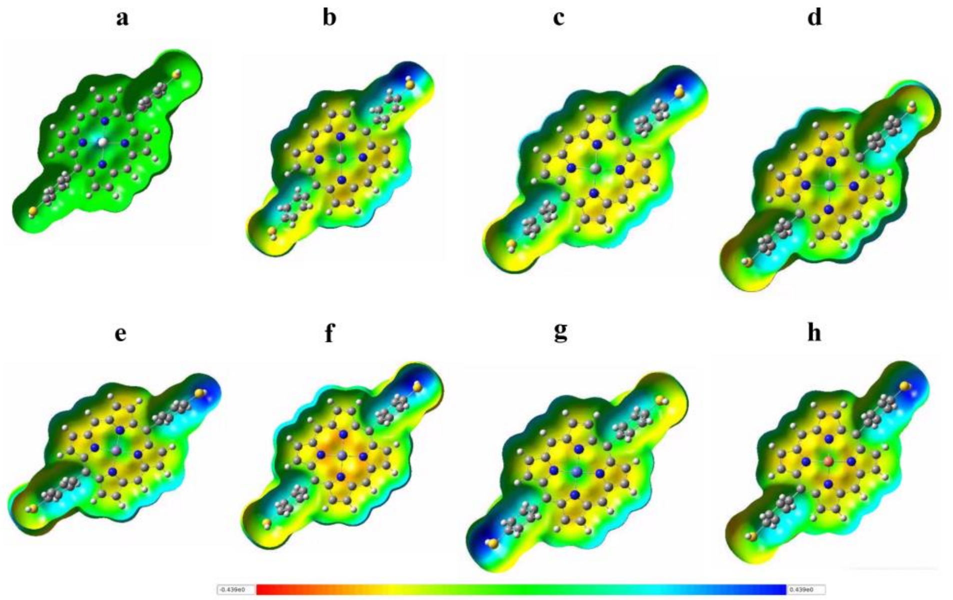

Figure S6. The Electrostatic potential map for MDPPSH (M = Sc--Cu), (ScDPPSH (a), TiDPPSH (b), VDPPSH (c), CrDPPSH (d), MnDPPSH (e), FeDPPSH (f), CoDPPSH (g), CuDPPSH (h) systems, respectively).

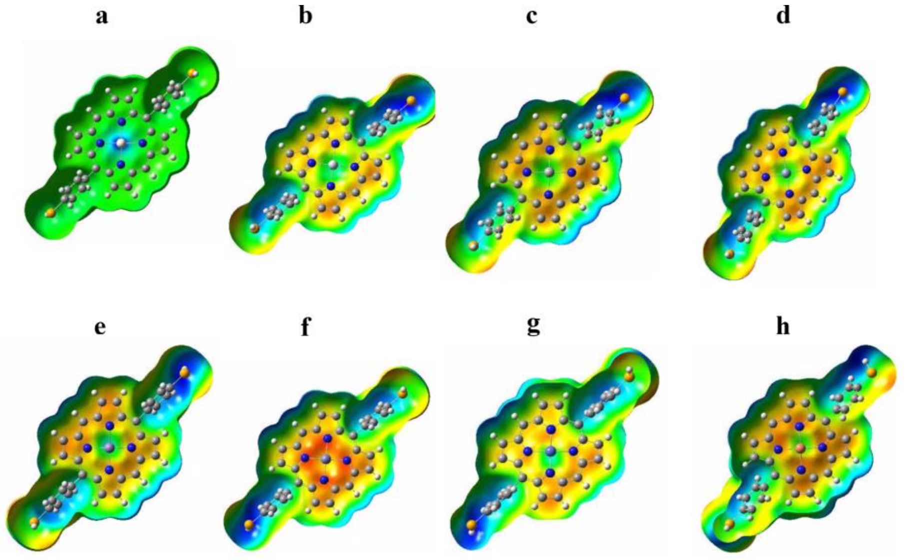

Figure S7. The Electrostatic potential map for MDPPSeH (M = Sc--Cu), (ScDPPSeH (a), TiDPPSeH (b), VDPPSeH (c), CrDPPSeH (d), MnDPPSeH (e), FeDPPSeH (f), CoDPPSeH (g), CuDPPSeH (h) systems, respectively).

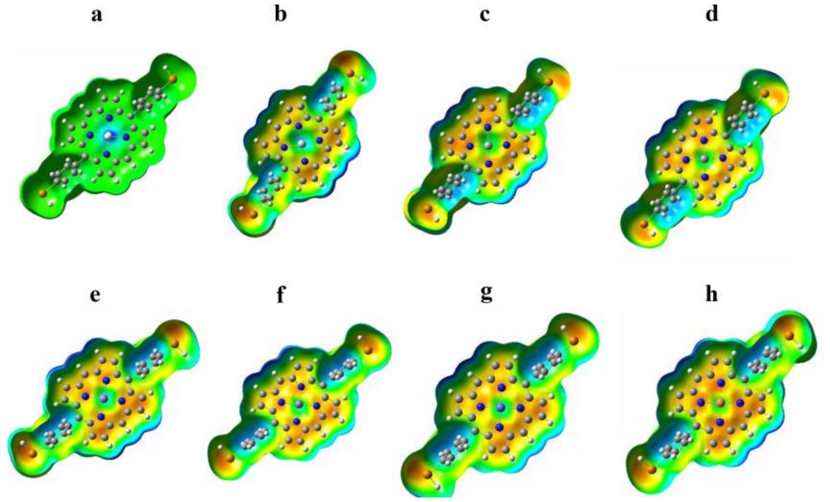

Figure S8. The Electrostatic potential map for MDPPTeH (M = Sc--Cu), (ScDPPTeH (a), TiDPPTeH (b), VDPPTeH (c), CrDPPTeH (d), MnDPPTeH (e), FeDPPTeH (f), CoDPPTeH (g), CuDPPTeH (h) systems, respectively).

References

1. Pearson, R. G. The electronic chemical potential and chemical hardness. *J. Mol. Struc. THEOCHEM* 1992, *255*, 261–270.
2. Bhatia, M. An overview of conceptual-DFT based insights into global chemical reactivity of volatile sulfur compounds (VSCs). *Comput. Toxicol.* 2023, 100295.
3. Padmanabhan, J.; Parthasarathi, R.; Elango, M.; Subramanian, V.; Krishnamoorthy, B.; Gutierrez-Oliva, S.; Toro-Labbé, A.; Roy, D.; Chattaraj, P. Multiphilic descriptor for chemical reactivity and selectivity. *J. Phys. Chem. A* 2007, *111*, 9130–9138.
4. Bashir, B.; Alotaibi, M. M.; Clayborne, A. Z. Computational investigation of structural, electronic, and spectroscopic properties of Ni and Zn metalloporphyrins with varying anchoring groups. *J. Chem. Phys.* 2024, 160.
5. Ratti, C.; Richard, P.; Tabard, A.; Guilard, R. Synthesis and characterization of a new series of titanium (IV) porphyrins coordinated to a disulphur or a diselenium ligand. *J. Chem. Soc., Chem. Commun.* 1989, 69–70.
6. Scheidt, W. R.; Reed, C. A. Stereochemistry of the toluene solvate of. alpha.,. beta.,. gamma.,. delta.-tetraphenylporphinatochromium (II). *Inorg. Chem.* 1978, *17*, 710–714.
7. Kirner, J. F.; Reed, C. A.; Scheidt, W. R. Stereochemistry of manganese porphyrins. 2. The toluene solvate of. alpha.,. beta.,. gamma.,. delta.-tetraphenylporphinatomanganese (II) at 20 and-175. degree. C. *J. Am. Chem. Soc.* 1977, *99*, 1093–1101.
8. Wondimagegn, T.; Rauk, A. The structures and stabilities of the complexes of biologically available ligands with Fe (III)− porphine: an ab initio study. *J. Phys. Chem. B* 2011, *115*, 569–579.
9. Collman, J. P.; Hoard, J.; Kim, N.; Lang, G.; Reed, C. A. Synthesis, stereochemistry, and structure-related properties of. alpha.,. beta.,. gamma.,. delta.-tetraphenylporphinatoiron (II). *J. Am. Chem. Soc.* 1975, *97*, 2676–2681.

10. Scheidt, W. R. Stereochemistry of low-spin cobalt porphyrins. III. Crystal structure and molecular stereochemistry of bis (piperidine)-. alpha.,. beta.,. gamma.,. delta.-tetraphenylporphinatocobalt (II). *J. Am. Chem. Soc.* 1974, *96*, 84–89.
11. Fleischer, E. B.; Miller, C. K.; Webb, L. E. Crystal and molecular structures of some metal tetraphenylporphines. *J. Am. Chem. Soc.* 1964, *86*, 2342–2347.
12. Gaussian 16, Revision C.02, Frisch, M. J.; Trucks, G. W.; Schlegel, H. B.; Scuseria, G. E.; Robb, M. A.; Cheeseman, J. R.; Scalmani, G.; Barone, V.; Petersson, G. A.; Nakatsuji, H.; Li, X.; Caricato, M.; Marenich, A. V.; Bloino, J.; Janesko, B. G.; Gomperts, R.; Mennucci, B.; Hratchian, H. P.; Ortiz, J. V.; Izmaylov, A. F.; Sonnenberg, J. L.; Williams-Young, D.; Ding, F.; Lipparini, F.; Egidi, F.; Goings, J.; Peng, B.; Petrone, A.; Henderson, T.; Ranasinghe, D.; Zakrzewski, V. G.; Gao, J.; Rega, N.; Zheng, G.; Liang, W.; Hada, M.; Ehara, M.; Toyota, K.; Fukuda, R.; Hasegawa, J.; Ishida, M.; Nakajima, T.; Honda, Y.; Kitao, O.; Nakai, H.; Vreven, T.; Throssell, K.; Montgomery, J. A., Jr.; Peralta, J. E.; Ogliaro, F.; Bearpark, M. J.; Heyd, J. J.; Brothers, E. N.; Kudin, K. N.; Staroverov, V. N.; Keith, T. A.; Kobayashi, R.; Normand, J.; Raghavachari, K.; Rendell, A. P.; Burant, J. C.; Iyengar, S. S.; Tomasi, J.; Cossi, M.; Millam, J. M.; Klene, M.; Adamo, C.; Cammi, R.; Ochterski, J. W.; Martin, R. L.; Morokuma, K.; Farkas, O.; Foresman, J. B.; Fox, D. J. Gaussian, Inc., Wallingford, CT, USA, 2016.
